# Enhancement of Biodiesel Production from Marine Alga, *Scenedesmus* sp. through *In Situ* Transesterification Process Associated with Acidic Catalyst

**DOI:** 10.1155/2014/391542

**Published:** 2014-02-13

**Authors:** Ga Vin Kim, WoonYong Choi, DoHyung Kang, ShinYoung Lee, HyeonYong Lee

**Affiliations:** ^1^Department of Bioengineering and Technology, College of Engineering, Kangwon National University, Chuncheon 200-701, Republic of Korea; ^2^Department of Medical Biomaterial Engineering, Kangwon National University, Chuncheon 200-701, Republic of Korea; ^3^Korea Institute of Ocean Science & Technology (KIOST), Gyeonggi-do, Ansan-si, P.O. Box 29, Seoul 426-744, Republic of Korea; ^4^Department of Food Science and Engineering, Seowon University, Cheongju, Chungbuk 361-742, Republic of Korea

## Abstract

The aim of this study was to increase the yield of biodiesel produced by *Scenedesmus* sp. through *in situ* transesterification by optimizing various process parameters. Based on the orthogonal matrix analysis for the acidic catalyst, the effects of the factors decreased in the order of reaction temperature (47.5%) > solvent quantity (26.7%) > reaction time (17.5%) > catalyst amount (8.3%). Based on a Taguchi analysis, the effects of the factors decreased in the order of solvent ratio (34.36%) > catalyst (28.62%) > time (19.72%) > temperature (17.32%). The overall biodiesel production appeared to be better using NaOH as an alkaline catalyst rather than using H_2_SO_4_ in an acidic process, at 55.07 ± 2.18% (based on lipid weight) versus 48.41 ± 0.21%. However, in considering the purified biodiesel, it was found that the acidic catalyst was approximately 2.5 times more efficient than the alkaline catalyst under the following optimal conditions: temperature of 70°C (level 2), reaction time of 10 hrs (level 2), catalyst amount of 5% (level 3), and biomass to solvent ratio of 1 : 15 (level 2), respectively. These results clearly demonstrated that the acidic solvent, which combined oil extraction with *in situ* transesterification, was an effective catalyst for the production of high-quantity, high-quality biodiesel from a *Scenedesmus* sp.

## 1. Introduction

Biodiesel is an alternative renewable fuel that is derived from vegetable oils, animal fats, spent frying oils, and microbial oils. This fuel can replace petroleum-based fuels and can be used in all types of the currently available diesel engines without them being modified [1, 2].

Extensive investigation of biodiesel manufacturing has been conducted using oil-like materials, such as vegetable oils, animal fats, and used frying oils, which are limited by their availability, and the prices of these materials are more greatly sensitive to the industrial demands for oil. The limited inventories also created a fuel versus food issue that requires consideration of non-food-related feedstock. Therefore, some attempts have been made to produce biodiesel from nonedible resources such as spent frying oils, greases, and *Jatropha*. However, a major criticism of large-scale fuel production from nonedible resources is that vast areas of farmland or native habitats will be consumed, and food prices will increase. Because of these problems, microalgae are currently considered one of the most promising resources for biodiesel production [3–6].

Microalgae exhibit a high photosynthetic efficiency and a strong capacity to adapt to the environment (e.g., high salinity, heavy metal ion content, presence of toxicants, and high CO_2_ concentration). Moreover, microalgae can be grown in various climates and on nonarable land, including marginal areas that are unsuitable for agricultural purposes (e.g., desert and seashore lands). The growth of microalgae in liquid medium can be controlled easily, and they can be cultured in nonpotable water. Moreover, most microalgae (e.g., *Scenedesmus* and *Chlorella*) have very short cell cycles (less than 24 hours) and high oil productivity per hectare. Therefore, microalgae have been predicted to be a new biofuel source that is renewable and is environmentally and economically sustainable [3, 6–9].

Many species of microalgae suitable for producing fuel oil have been identified, that is, *Chlorella*, *Scenedesmus*, *Spirulina*, and *Nostoc* spp. Supplementation of iron to the growth medium under nitrate limitation was found to raise the crude lipid content of *Chlorella* to ~56.6% (dry cell weight). The lipid content of *Scenedesmus obliquus* reached up to ~58.3% (dry cell weight) when lower nitrogen-supplemented medium was utilized [10–13]. In our preliminary studies [14, 15], *Scenedesmus* sp. was shown to be an appropriate source for biodiesel by determining its lipid composition and extraction yield. Therefore, we decided to use this species as a biodiesel feedstock in the present study.

FAMEs (fatty acid methyl esters) are produced via a transesterification process that involves a chemical reaction between triglycerides and an alcohol, generally methanol, in the presence of a catalyst [16, 17]. This process is the one that is most commonly used to produce industrial biodiesel due to the low cost of the catalyst, its utility in mass production, and its high productivity. However, this method often produces a large amount of hazardous solvent waste and is generally cumbersome. This transesterification process requires preextracting the oil from the raw materials. Automated extraction equipment has been successfully developed, but it requires a longer extraction process [6, 18, 19].

Recently, an *in situ *transesterification (or direct transesterification) method that may overcome these limitations has attracted attention. *In situ *transesterification differs from the conventional method because the oil-bearing material directly contacts the acidified or alkalized alcohol instead of preextracted oil reacting with the alcohol. Thus, the extraction and transesterification processes occur in the same step. The alcohol acts as both an extraction solvent and an esterification reagent, which could reduce the production time associated with preextracting the oil and maximize the fatty acid ester yield. By using such reagents, the amount of solvents and the processing period are reduced and the problems of waste disposal are avoided [17, 20, 21].

The most conventional approach to experiment design is to optimize one variable (or one factor) at a time in single-response problems. An increasing number of factors increases the number of design experiments. However, the increasing complexity of product design is such that at least two quality characteristics are frequently simultaneously considered to improve the product quality. Moreover, correlations among multiple responses always exist and may generate conflicts among the optimal parameter settings. Hence, simultaneously optimizing a multiresponse problem is important [22].

The Taguchi method is a powerful experimental design tool that provides a simple, effective, systematic approach to determining the optimal parameters. Furthermore, this approach requires minimum experimental cost and it efficiently reduces the effect of the sources of variation [23]. The design of experiments (DOE) methodology of the Taguchi orthogonal array (OA), a factorial-based approach, has recently become exceeding important due to its application in optimizing biochemical processes [24].

In this biodiesel research study, we investigated using microalgae as the feedstock for biodiesel production. A microalgal *Scenedesmus* sp. was selected for biodiesel production through *in situ *transesterification via acidic catalysis and the synthesis of this biofuel was optimized using the Taguchi method.

## 2. Materials and Methods

### 2.1. Materials

Freeze-dried microalgal cells were provided by KORDI (Korea Ocean Research & Development Institute). The microalga was cultured under controlled conditions, and the algal biomass was harvested by centrifugation at 3000 rpm for 10 min. The cells were then freeze-dried, ground (100 mesh), and stored in the freezer. The freeze-dried powder was kept under desiccation of anhydrous sodium sulfate (Daejung Co., Ltd, Shi heung, Korea) overnight, before use.

### 2.2. *In Situ* Transesterification via Alkaline or Acidic Catalysis

**Figure 1:**
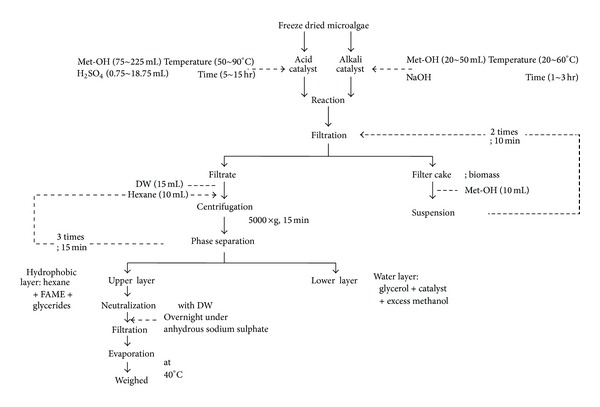
A schematic diagram of the *in situ *acidic and alkaline transesterification reactions.

The *in situ *transesterification process following the method of Ehimen et al. [25] was conducted at the laboratory scale. The alkaline process experiments were conducted under the following conditions: reaction time of 1 to 3 hrs, reaction temperature of 40 to 60°C, quantity of the reacting alcohol (methanol, 99.5%, Daejung Co. Ltd, Shi Heung, Korea) of 20 to 50 mL, and concentrations of sodium hydroxide of 0.5 to 1.5% (w/w of lipid). The reactor had a capacity of 250 mL, and it was immersed in a water bath to maintain the reaction temperature at the desired level. The reaction was conducted under reflux conditions to minimize the solvent loss. The acidic process experiments were conducted under the following conditions: reaction time of 5 to 15 hrs, reaction temperature of 50 to 90°C, biomass to solvent ratio of 5 : 1 to 25 : 1 (v/w), and concentrations of sulfuric acid of 1 to 5% (v/v of solvent). The reactor capacity was 500 mL. A freeze-dried algal biomass (15 g) was mixed with methanol in which sulfuric acid had been dissolved. The scheme of the *in situ *transesterification reaction and product purification steps are shown in Figure 1. Additionally, a freeze-dried algal biomass (5 g) was mixed with methanol in which 0.5% NaOH had been dissolved as an alkaline catalyst. Thus, the acidic catalyst of 5% H_2_SO_4_ was replaced with sodium hydroxide. The reactor containing the reaction mixture was heated and then maintained at the temperature of interest for the specified periods. After the *in situ *transesterification step, the reaction flask was allowed to stand for 1 hour to allow its contents to settle. The reaction mixture was filtered (Advantec No. 2, Toyo Roshi Inc., Tokyo, Japan) and the residues were washed twice by resuspension in methanol (10 mL) for 10 min to recover any traces of FAME products from the residues. Distilled water (15 mL) was added to the filtrate to facilitate the separation of the hydrophilic components of the extract. This mixture was centrifuged (5000 ×g) for 15 min and then transferred to a separation funnel. Further extraction of the FAME products was achieved by three times for 15 min extractions using 10 mL of hexane. The pooled hexane layer was washed with water (to remove the residual traces of the acidic catalyst and methanol), separated, and then dried over anhydrous sodium sulfate overnight. The extracted solvent layer was filtered (Advantec No. 2, Toyo Roshi Inc., Tokyo, Japan), and its volume was recorded. An aliquot of the extraction solvent (10 mL) containing the FAMEs (crude biodiesel) was transferred to a preweighed glass vial evaporated (40~45°C) for 15 min using a vacuum evaporator (EYELA, Digital Water Bath SB-1000, Japan) and then cooled within a desiccator for 30 min. The mass of the biodiesel was determined gravimetrically, in duplicate. All of the stirred reactions were conducted using a magnetic stirrer system with a constant rotation speed of 500 rpm. The biodiesel yield from the algae oil was calculated using (1) as follows:

(1) Biodiesel  yield (%) =Algae  mass (g)×Lipid  content (%)Weight  of  biodiesel (FAME) (g),

in which the amount of FAMEs at each step was determined by collecting them from a TLC plate and weighing them.

### 2.3. Thin Layer Chromatography (TLC) Analysis

After transesterification, the FAMEs (fatty acid methyl esters (FAME)) and residual triacylglycerides (TAGs) were measured using 0.25 mm thick silica gel G-60 TLC plates (Merck, Darmstadt, Germany). The detailed method was as follows: the elution solvent was n-hexane : diethyl ether (90 : 10, v/v) and after full development to the detection of the FAME spots, the spots were visualized using an iodine vapor and spraying the plates with 10% phosphomolybdic acid (98% purity, Daejung Co., Ltd, Shi Heung, Korea) in ethanol, and then drying them in the oven at 105°C [26]. The mono-, di-, and triglyceride mixture (TAG STD, Supelco, Bellefonte, PA, USA) and commercial biodiesel (FAME, S Company Houston, TX, USA) were used as standards [26]. Additionally, the pigments that remained in the FAME fraction were also separated by TLC, using the same 0.25 mm thick silica gel G-60 plates (Merck, Darmstadt, Germany) that were developed using petroleum ether (99.5%, Daejung Co., Ltd, Shi Heung, Korea) : cyclo hexane (%, Wako, Osaka, Japan) : ethyl acetate (99%, Daejung Co., Ltd, Shi Heung, Korea) : acetone (99.5%, Daejung Co., Ltd, Shi Heung, Korea) : methanol (99.5%, Daejung Co., Ltd, Shi Heung, Korea) (60 : 16 : 10 : 10 : 4, v/v) [27].

### 2.4. Purification of FAMEs

The FAMEs of the crude biodiesel were purified using silica gel column chromatography. The crude biodiesel was applied to a silica gel chromatography column. The column had been prepared using the previously described slurry of silica gel (18 g) (0.063–0.2 mm, Merck, Darmstadt, Germany) in hexane (300 mL). The n-hexane level was lowered until it was 1 cm above that of the stationary phase. The extracted algal oil was added to the column and eluted using n-hexane : EtOAC (25 : 1, v/v) at a flow rate of 3 mL/min. The FAME fraction had a deep yellow color [28, 29].

### 2.5. Design of the Experiments: Taguchi Method

Experiments were designed to determine the effects of four factors, the reaction temperature, the reaction period, the amount of catalyst (H_2_SO_4_), and the biomass to solvent ratio. Nine experiments were designed using the Taguchi method with an L9 three-level-four-factor array [34].

### 2.6. Analysis of the FAME Fatty Acid Profiles

To determine the fatty acid composition of the FAMEs, gas chromatography (GC, HP 6890 series, Victoria, Australia) was conducted using the following method: 1 *μ*L of FAME that was obtained using *in situ* transesterification via alkaline or acidic catalysis was injected into a GC with a flame ionization detector (FID); an SP-2560 column (100 m × 0.25 mm × 0.2 *μ*m, #24056, Supelco); and an oven temperature that increased at 4°C/min from 100°C to 240°C. The pouring and detector temperatures were set at 250°C and 280°C, respectively, the flow volume was 1.0 mL, and the split ratio was 50 : 1 [30].

### 2.7. Statistical Analysis

The data are expressed as the mean values ± SDs (standard deviation) and the mean values are the averages of five test results per experiment. The data were analyzed using the Student's *t*-test (SAS 9.1, SAS, Cary, NC, USA). The experiments were repeated at least three times to confirm the results. The data were analyzed using an analysis of variance, and the mean values were considered significantly different at *P* < 0.05. The optimal extraction condition was determined using regression analysis.

## 3. Results and Discussion

### 3.1. Optimization of Crude Biodiesel Production Using an Alkaline or Acidic Catalyst

**Figure 2:**
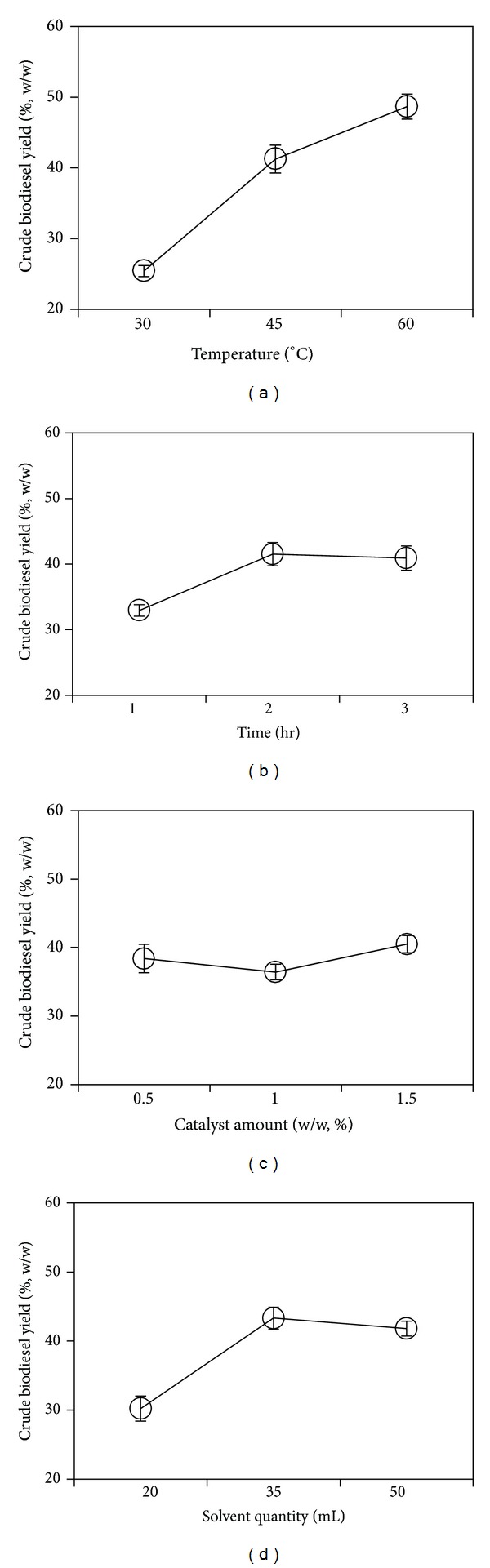
Main effects plot for the reaction factors in *in situ *transesterification via alkali catalysis.

**Figure 3:**
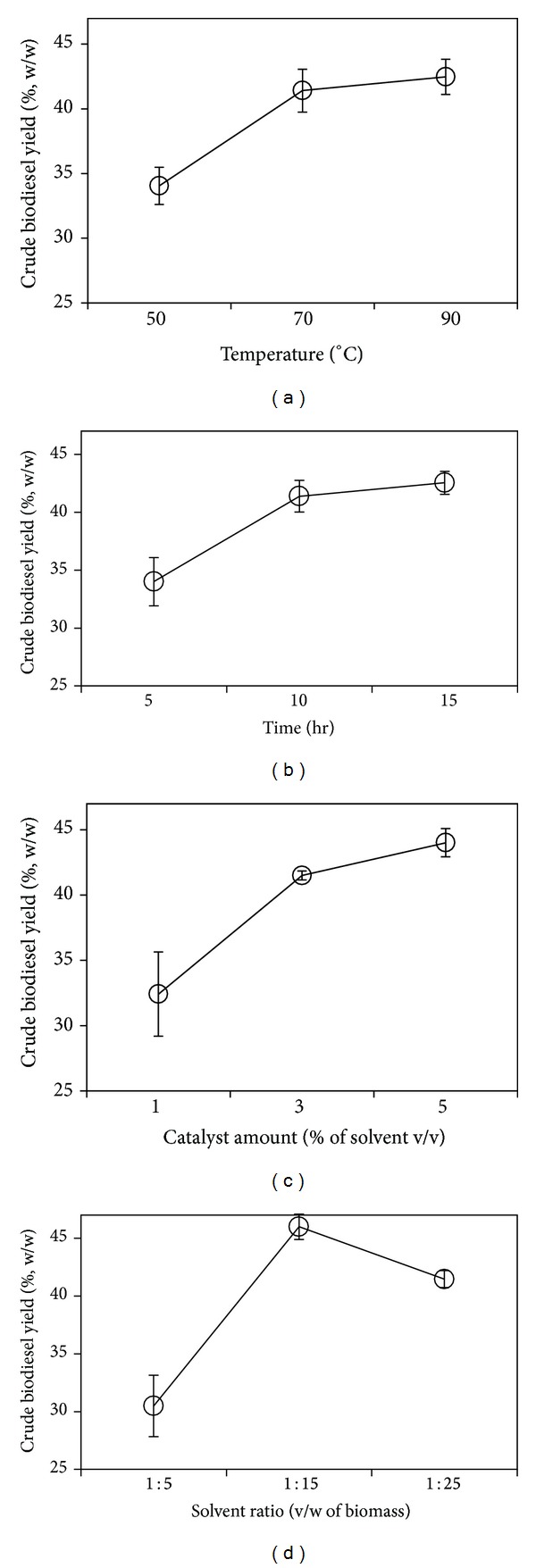
Main effects plot for the reaction factors in *in situ* transesterification via acidic catalysis.

For conventional two-step industrial biodiesel production, methanol is most often used as the reaction solvent with an alkaline catalyst for transesterification because it has several advantages over the simultaneous separation of glycerol, a high product yield, and low price [31], as well as mild processing conditions, short reaction times, and some other economic benefits [32–34]. Rather than this conventional transesterification process, *in situ* transesterification with an alkaline catalyst could be used; however, for the production of biodiesel from microalgae, an alkaline catalyst would not be suitable for the *in situ* transesterification process, due to the characteristically high FFA (free fatty acid) content of the microalgal lipids. *In situ* transesterification of oils containing high concentrations of FFA would result in a partial saponification reaction, leading to soap formation [25]. Soaps can cause the formation of emulsions, which create difficulties in the downstream recovery and purification of the biodiesel [35]. Using inorganic acids, such as hydrochloric acid and sulfuric acid, as reaction catalysts was therefore considered for microalgal lipid transesterification, due to their insensitivity to the FFA content of this lipid feedstock. Consequently, using acidic catalysis facilitates both the biodiesel producing transesterification and esterification reactions [25]. Studies of the catalytic activities of HCl and H_2_SO_4_ in the transesterification of *Thevetia peruviana* seed oil, cotton seed oil, and vegetable oil, among others, showed that H_2_SO_4_ exhibited better catalytic activity than HCl [36, 37]. Therefore, for *in situ* transesterification using an alkaline or acidic catalyst, several reaction parameters should be considered such as the reaction temperature, the reaction time, the amount of catalyst, the amount of solvent, the water content, and the agitation speed [36, 37]. To obtain the highest yield of biodiesel, these variables were optimized using the Taguchi methods [38], employing an L9 three-level-four-factor orthogonal array matrix [34]; nine experiments were designed as shown in Tables 1 and 3 for an alkaline or an acidic catalyst, respectively. Using an alkaline catalyst, as shown in Table 1, the biodiesel yield was the lowest and the highest in experiment number 1 and experiment number 8, respectively. Depending on the level of each factor, the yields changed from 11.63 ± 1.06 to 55.07 ± 2.18% and the average yield was 38.45 ± 1.50%. In each factor level (*A*_*i*_) of the yield (*K*_*i*_^*A*^) and at each *K*_*i*_^*A*^ value per unit level, the *k*_*i*_^*A*^ ( = *K*_*i*_^*A*^/3) were calculated from the difference (*R* value) between the maximum *K*_*i*_^*A*^ and minimum *K*_*i*_^*A*^. However, as shown in Table 3, the lowest and highest yields of crude biodiesel were obtained in experiment number 1 and experiment number 7, respectively. Depending on the level of each factor, the yields were changed from 13.03 ± 3.07 to 48.40 ± 0.51% and the average yield was 39.27 ± 0.94%. The factor effects decreased in the order of biomass to solvent ratio (34.36%) > catalyst amount (28.62%) > reaction time (19.72%) > reaction temperature (17.32%). From these factors, the size of the effect of the test parameters could be calculated, as shown in Tables 2 and 4.  Table 2 shows the effect of the reaction factors on the mean response using the alkaline catalyst. The effect refers to the average value of the crude biodiesel yield for each factor at the different levels. Thus, the average value for each factor at each of the three levels was calculated and plotted (Figure 2). Figure 2 shows the mean effects plot for the crude biodiesel yield of *in situ* transesterification via alkaline catalysis. The dots in the reaction time, amount of catalyst, and solvent quantity show the fluctuations in the biodiesel yield. The lowest biodiesel yield was obtained at the lowest reaction temperature; increasing the reaction temperature resulted in a higher biodiesel yield. The effects of the factors decreased in the order of reaction temperature (47.5%) > solvent quantity (26.7%) > reaction time (17.5%) > catalyst amount (8.3%). The optimal levels of each factor were obtained from the value of *k*_*i*_^*A*^, and these results lead to the conclusion that the factors that provided the maximal yield were a reaction temperature of 60°C, a solvent quantity of 35 mL, a reaction time of 2 hrs, and an amount of catalyst of 1.5% (based on the lipid weight). However, the effects of the reaction time and the amount of catalyst were weaker than that of the other factors. Therefore, the levels of the recommended factors were as follows: reaction temperature of 60°C (level 3), a solvent quantity of 35 mL (level 2), reaction time of 2 hrs (level 2), and an amount of catalyst of 0.5% (level 1). The theoretical biodiesel yield of 55.07 ± 2.18% (*P* < 0.05) could be obtained using these optimal conditions.  Figure 3 shows the mean effects plot for the crude biodiesel yield from *in situ* transesterification via acidic catalysis. The dots in biomass to solvent ratio showed the fluctuations of the biodiesel yield. When the levels of the reaction temperature, reaction time, and amount of catalyst increased, a higher biodiesel yield was obtained. The lowest biodiesel yield was obtained at the lowest biomass to solvent ratio, and the highest biodiesel yield was obtained at the middle level of the biomass to solvent ratio. The optimal levels of each factor were obtained from the value of *k*_*i*_^*A*^, and these results lead to the conclusion that the factors that provided the maximum yield were a reaction temperature of 90°C, a reaction time of 15 hrs, an amount of catalyst of 5% (base on solvent), and a biomass to solvent ratio of 1 : 15 (w/v). However, the time and temperature effects were lower than that of the other factors, and these conditions were not favorable for biodiesel production. A high reaction temperature increases the cost of biodiesel production [16]. A long reaction time also increases the cost of production [39]. Therefore, a reaction temperature of 70°C (level 2), a reaction time of 10 hrs (level 2), an amount of catalyst of 5% (level 3), and a biomass to solvent ratio of 1 : 15 (level 2) were selected as the optimal conditions. A crude biodiesel yield of 48.41 ± 0.21% (*P* < 0.05) could be obtained under these optimal conditions. The optimal conditions provided almost the same yield of biodiesel as the maximal conditions.

### 3.2. Identification and Purification of FAMEs

**Figure 4:**
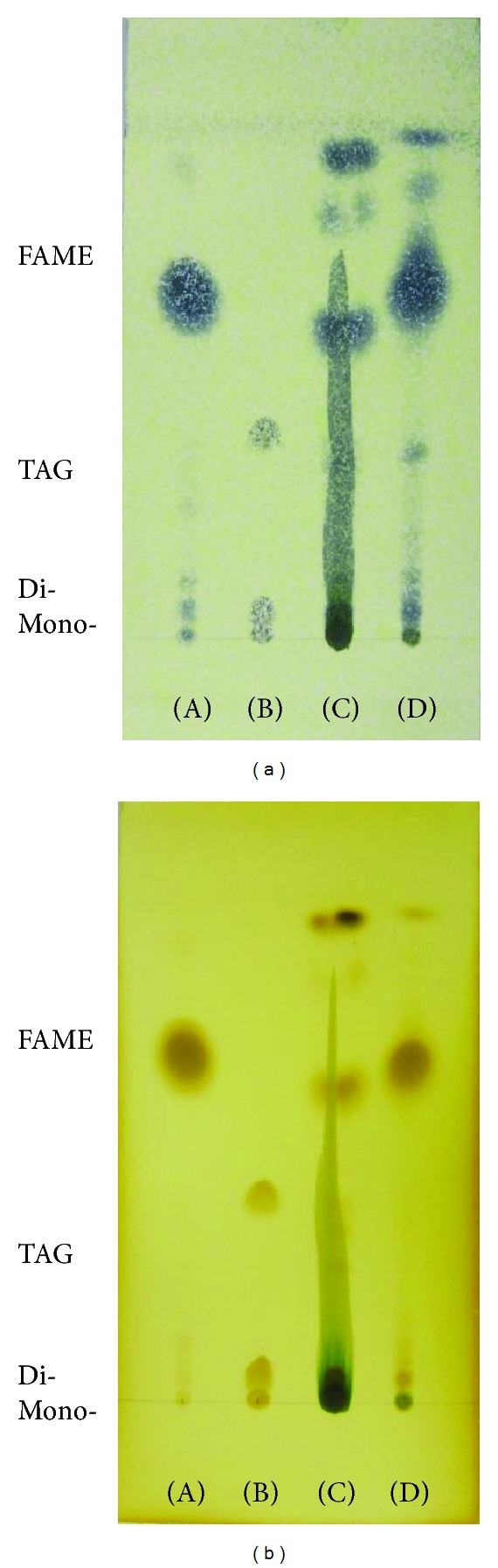
Thin layer chromatograms of crude FAMEs on HPTLC plates that were developed using n-hexane : ether (90 : 10, v/v) and visualized using (a) 10% phosphomolybdic acid in ethanol and (b) iodine vapor. (A) FAMEs (standard); (B) TAGs (standard); (C) *in situ *transesterification via alkaline catalysis; (D) *in situ *transesterification via acidic catalysis.

**Figure 5:**
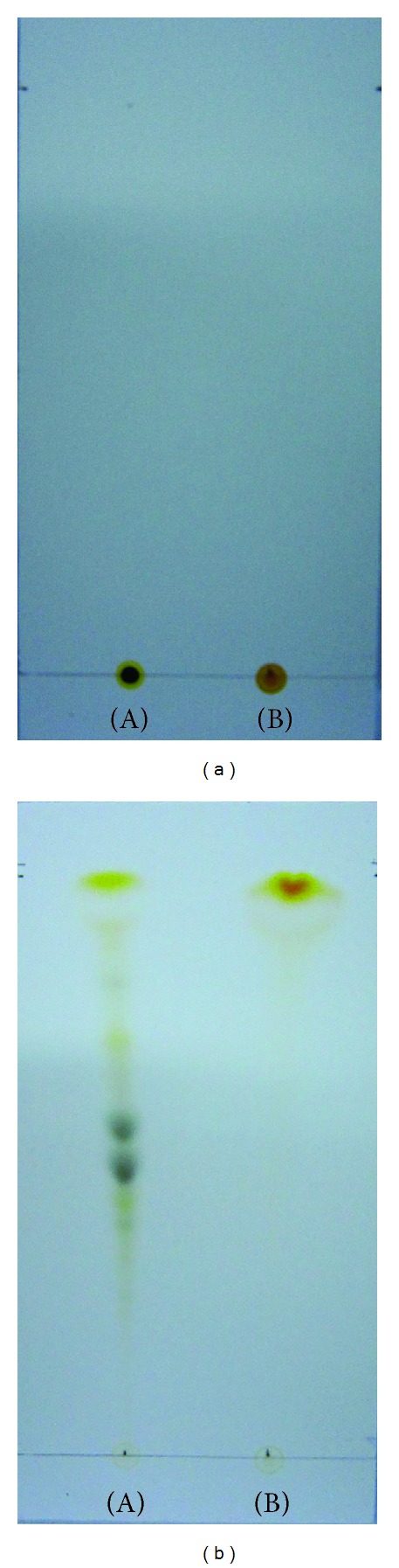
Results of separating the pigment fractions using TLC (20 times dilution with hexane). (a) Before development; (b) after development; (A) crude biodiesel; (B) purified biodiesel.

**Figure 6:**
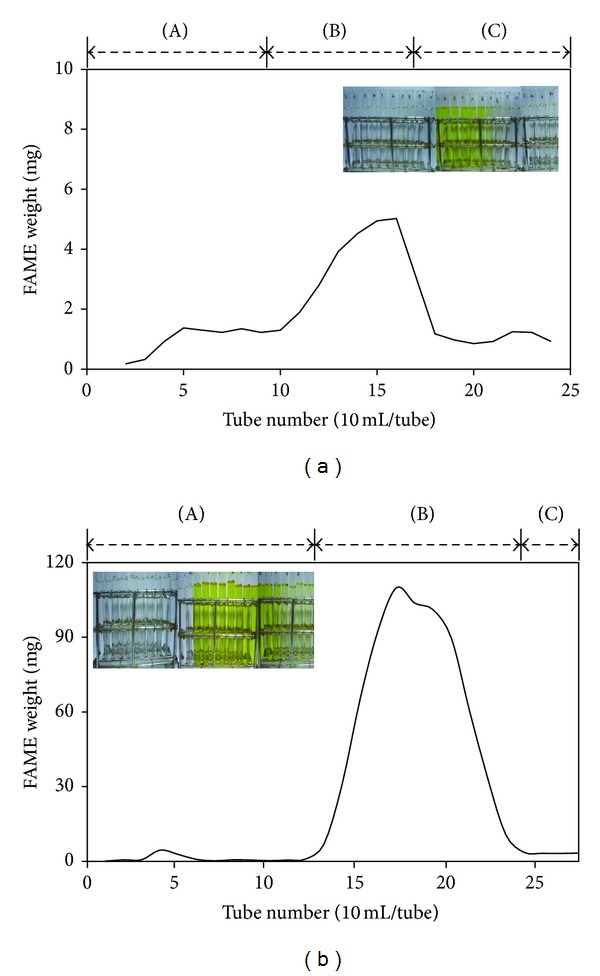
Column chromatographic separation of FAME using an n-hexane : acetone (98.5 : 1.5, v/v) solution from (a) alkali- and (b) acid-catalyzed biodiesels.

**Figure 7:**
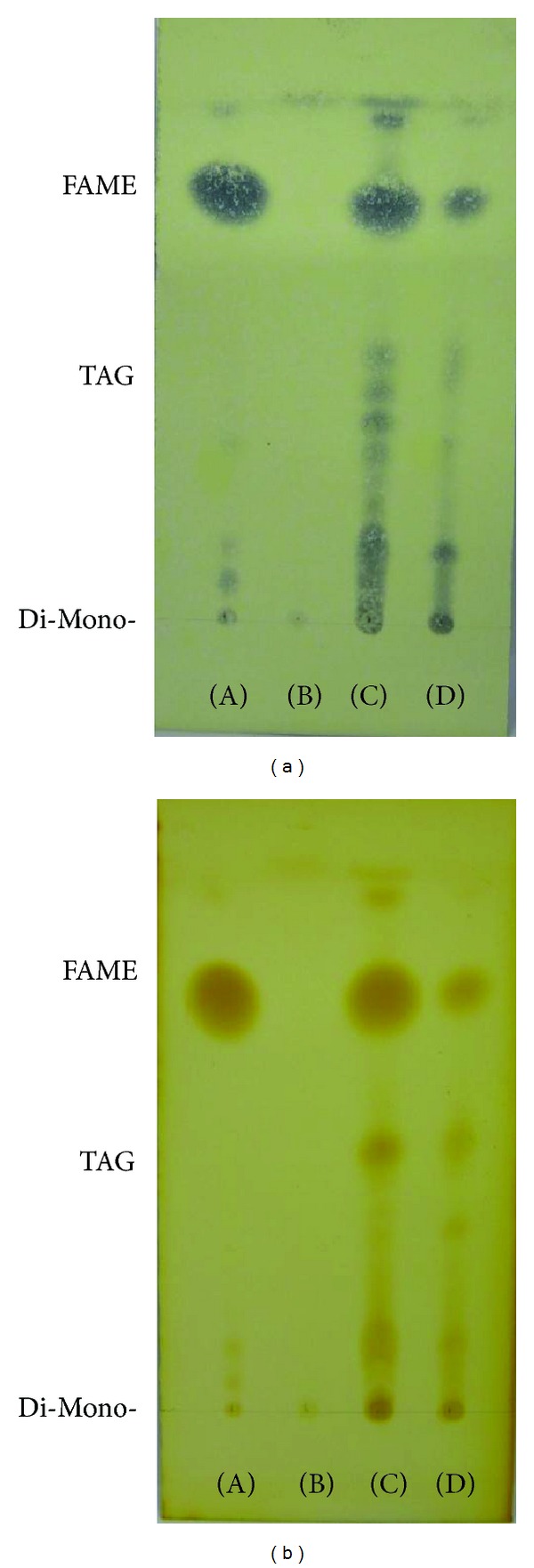
Thin layer chromatograms of the FAME fraction on HPTLC plates that were developed using n-hexane : ether (90 : 10, v/v) and visualized using (a) 10% phosphomolybdic acid in ethanol (b) and iodine vapor. (A) FAME STD; (B) Fraction A (tube numbers: 1–10); (C) Fraction B (tube numbers: 11–16); (D) Fraction C (tube numbers: 17–24).

**Figure 8:**
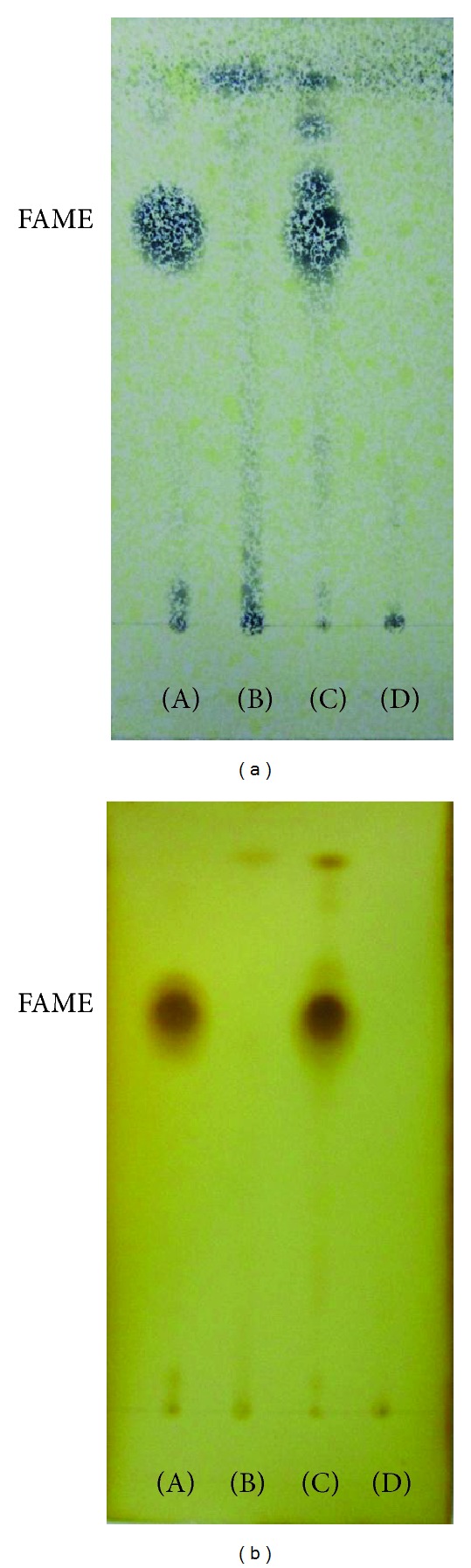
Thin layer chromatograms of the FAME fractions on HPTLC plates that were developed using n-hexane : ether (90 : 10, v/v) and visualized using (a) 10% phosphomolybdic acid in ethanol and in (b) iodine vapor. (A) FAME STD; (B) fraction A (tube numbers: 1–12); (C) fraction B (tube numbers: 13–23); (D) fraction C (tube numbers: 24–27).

Thin layer chromatography (TLC) is one of the simplest and most widely used methods for separating mixtures for analysis. In this study, it was used to monitor the conversion of the algal lipids to fatty acid methyl esters (FAMEs, namely, biodiesel). The TLC results demonstrated the presence of FAME, TAG, and free fatty acids. The TAG standard was a mixture of mono-, di-, and triglycerides, and the FAME standard was a commercial biodiesel (as a comparative standard). Each standard was eluted using n-hexane. The retention factors for methyl ester and triglycerides were calculated as *R*_*f*_, Me = 0.7–0.73 and *R*_*f*_, TAG = 0.41–0.45, respectively. The compound with the larger *R*_*f*_ is less polar because it interacts less strongly with the polar adsorbent on the TLC plate. Here, the methyl esters and triglycerides exhibited the characteristics of high nonpolarity. As shown in Figure 4, after the TLC plates had been developed, a dark trace appeared only in the alkaline catalyzed biodiesel, which was green before the visualization reaction was performed. The photosynthetic microalga *Scenedesmus* sp. synthesizes pigments such as chlorophylls and carotenoids. The dark green trace was considered to contain pigments. TLC was used to identify the pigments, which consisted of chlorophyll a, *β*-carotene, pheophytin a and b, and other substances (Figure 5) [27]. Silica gel column chromatography of crude alkali-catalyzed biodiesel was used to fractionate the pigments and the FAMEs. The column was eluted with n-hexane : acetone (98.5 : 1.5, v/v). As shown in Figure 6(a), three fractions were obtained from the crude alkali-catalyzed biodiesel. The eluted materials were collected in individual vials and were fractionated depending on the color and weight difference between these vials. Fraction A (tube numbers 1–10) was colorless and transparent, but the colors of fraction B (tube numbers 11–16) and fraction C (tube numbers 17–24) were deep yellow and pale yellow, respectively. The green-pigmented materials adsorbed to the silica gel inside the column. The organic solvent of each vial was evaporated in a dry oven at 40°C for a few days, and then the vials were weighed. The materials inside the vials were redissolved in hexane and were then collected according to fractionation (fractions A, B, and C). Each of the fractions was analyzed using TLC, and the results are shown in Figure 7. TLC on silica gel plate demonstrated the presence of methyl esters, tri-, di-, and monoglycerides and fatty acids in the alkali-catalyzed biodiesel (Figure 7). FAMEs were observed in fractions B and C. Then, we conducted the same experiment using acid-catalyzed biodiesel. The color of fraction A (tube numbers 1–12) was colorless and transparent, but the colors of fraction B (tube numbers 13–23) and fraction C (tube numbers 24–27) were deep yellow and pale yellow (almost colorless), respectively (Figure 6(b)). The materials inside the vials were redissolved in hexane, and then each fraction was collected (fractions A, B, and C). Each fraction was analyzed using TLC, and the results are shown in Figure 8. FAMEs were observed in fraction B. The FAME fraction was purified using silica gel column chromatography, and the purified FAME contents were found to be 29.15% in the crude alkali-catalyzed biodiesel and 83.52% in the acid-catalyzed biodiesel. Approximately 70% of the alkali-catalyzed biodiesel and 16% of the acid-catalyzed biodiesel were found to contain pigments, which consisted of *β*-carotene, chlorophyll a, pheophytin a and b, and other substances (Figure 5). Therefore, the final purified biodiesel yields obtained from alkali- and acid-catalyzed biodiesel were estimated to be 16.05% and 40.43%, respectively. The yield of the acid-catalyzed biodiesel was approximately 2.5-fold higher than that of the alkali-catalyzed biodiesel. The yield from *in situ* transesterification using an acidic catalyst appeared to be higher than other reported data obtained using the conventional Taguchi method by 27.9~42.6% [40–42]. To evaluate the quality of the biodiesel obtained using these processes, the contents of the major fatty acid in the FAME profiles were estimated, as shown in Table 5. In general, there was not much difference in the fatty compositions of the acidic and alkaline catalyzed analytes; however, higher amounts of C_16 : 0_ and C_18 : 3_, the two major fatty acids among the FAMEs, were obtained using acidic catalysis, which implies that acidic catalysts can produce better quality biodiesel. These results indicated that the acidic catalyst was a more efficient catalyst than the alkaline catalyst for *in situ* transesterification.

## 4. Conclusion

To optimize *in situ* transesterification via alkaline or acidic catalysis for the production of biodiesel from *Scenedesmus* sp., several objective variables were analyzed using an L9 orthogonal matrix as follows: the reaction time and temperature and the amounts of catalyst and solvents [34]. In using an acidic catalyst in *in situ* transesterification, the effects of the factors decreased in the order of biomass to solvent ratio (34.36%) > catalyst amount (28.62%) > reaction time (19.72%) > reaction temperature (17.32%). The maximum yield of 48.41 ± 0.21% (*P* < 0.05) (based on lipid weight) was obtained at a biomass to solvent ratio of 1 : 15, H_2_SO_4_ at 5% (% of solvent, v/v), a reaction time of 10 hrs, and a reaction temperature of 70°C. For the alkaline catalyst, the effects of the factors decreased in the order of the reaction temperature (47.5%) > solvent quantity (26.7%) > reaction time (17.5%) > amount of catalyst (8.3%). The maximum biodiesel yield of 55.07 ± 2.18% (based on lipid weight) was obtained under the following conditions : solvent quantity of 50 mL, NaOH amount of 0.5% (based on the lipid weight), a reaction time of 2 hrs, and a reaction temperature of 60°C (*P* < 0.05). However, TLC analysis of the FAMEs showed that 48.41% of highly purified FAME was obtained through acidic catalysis, whereas 23.67% was obtained through alkaline catalysis, with less contamination by lipid pigments. It was also found that the quality of the biodiesel produced using the acidic catalyst was better than that produced using the alkaline catalyst by having higher amounts of C_16 : 0_ and C_18 : 3_. These results clearly indicated that an *in situ* transesterification process using an acidic catalyst should have an excellent potential for directly producing biodiesel from microalgae [43]. However, the entire process should be further developed to meet the economic feasibility required to scale up to industrial production. Nonetheless, these results could provide information for economically producing biofuels from *Scenedesmus* sp. microalga based on the quantity and quality of the algal biodiesel produced in this study.

## Figures and Tables

**Table:** (a)

Level	Temperature (°C)	Time (hr)	Catalyst amount (% of algal weight, w/w)	Solvent (mL)
	*A*	B	C	D
1	30	1	0.5	20
2	45	2	1	35
3	60	3	1.5	50

**Table:** (b)

Run number	A	B	C	D	Y (%)
1	1	1	1	1	11.63 ± 1.06
2	1	2	2	2	31.33 ± 0.79
3	1	3	3	3	33.29 ± 0.47
4	2	1	2	3	37.08 ± 0.58
5	2	2	3	1	38.13 ± 2.32
6	2	3	1	2	48.54 ± 2.99
7	3	1	3	2	50.07 ± 1.02
8	3	2	1	3	55.07 ± 2.18
9	3	3	2	1	40.89 ± 2.07

**Table 2:** Analysis of the *in situ *transesterification factors for the yield of crude biodiesel from *Scenedesmus *sp. using the orthogonal array design (% w/w, based on the lipid weight).

	*A*	B	C	D
*K*_1_	76.25 ± 2.3	98.78 ± 2.7	115.21 ± 6.2	90.65 ± 5.4
*K*_2_	123.75 ± 5.9	124.51 ± 5.3	109.31 ± 3.4	129.94 ± 4.8
*K*_3_	146.01 ± 5.3	122.72 ± 5.5	121.49 ± 3.8	125.42 ± 3.2

*k*_1_	25.42 ± 0.77	32.93 ± 0.88	38.4 ± 2.07	30.22 ± 1.82
*k*_2_	41.25 ± 1.96	41.5 ± 1.76	36.44 ± 1.14	43.31 ± 1.60
*k*_3_	48.67 ± 1.75	40.91 ± 1.84	40.5 ± 1.27	41.81 ± 1.08

*R*	23.25	8.58	4.06	13.09

Optimal level	3	2	3	2

*K*_*i*_^*A*^ = ∑ biodiesel yield at *A*_*i*_.****

*k*_*i*_^*A*^ = *K*_*i*_^*A*^/3.****

*R*_*i*_^*A*^ = max⁡{*k*_*i*_^*A*^} − min⁡{*k*_*i*_^*A*^}.

**Table:** (a)

Level	Temperature (°C)	Time (hr)	Catalyst amount (% of solvent, v/v)	Biomass to solvent ratio (mL, v/w)
	A	B	C	D
1	50	5	1	1 : 5
2	70	10	3	1 : 15
3	90	15	5	1 : 25

**Table:** (b)

Run number	A	B	C	*D*	*Y* (%)
1	1	1	1	1	13.03 ± 3.07
2	1	2	2	2	44.72 ± 0.18
3	1	3	3	3	44.13 ± 0.34
4	2	1	2	3	40.38 ± 0.36
5	2	2	3	1	39.36 ± 0.82
6	2	3	1	2	44.46 ± 1.08
7	3	1	3	2	48.40 ± 0.51
8	3	2	1	3	39.80 ± 1.65
9	3	3	2	1	39.07 ± 0.42

**Table 4:** Analysis of the *in situ *transesterification factors for the yield of crude biodiesel from *Scenedesmus *sp. using the orthogonal array design (% w/w, based on lipid weight).

	*A*	*B*	*C*	*D*
*K*_1_	102.13 ± 3.61	102.03 ± 4.46	97.26 ± 7.04	91.46 ± 4.71
*K*_2_	124.24 ± 2.00	124.14 ± 2.77	124.50 ± 1.33	137.97 ± 1.13
*K*_3_	127.42 ± 2.61	127.63 ± 0.99	132.04 ± 0.16	124.38 ± 2.38

*k*_1_	34.04 ± 1.20	34.01 ± 1.49	32.42 ± 2.35	30.49 ± 1.57
*k*_2_	41.41 ± 0.67	41.38 ± 0.92	41.50 ± 0.44	45.99 ± 0.38
*k*_3_	42.47 ± 0.87	42.54 ± 0.33	44.01 ± 0.05	41.46 ± 0.79

*R*	8.43 ± 0.24	8.54 ± 1.16	11.59 ± 2.40	15.50 ± 1.60

Optimal level	3	3	3	2

*K*_*i*_^*A*^ = ∑ biodiesel yield at *A*_*i*_ .****

*k*_*i*_^*A*^ = *K*_*i*_^*A*^/3 .****

*R*_*i*_^*A*^ = max⁡{*k*_*i*_^*A*^} − min⁡{*k*_*i*_^*A*^}.

**Table 5:** Comparison of fatty acids content in *Scenedesmus* sp. by *in situ *transesterification alkali and acid catalysis.

Catalyst	Fatty acids composition (%)						
	C_14 : 0_	C_16 : 0_	C_16 : 1_	C_18 : 0_	C_18 : 1_	C_18 : 2_	C_18 : 3_
Acid	0.43	33.21	0.69	1.70	0.67	11.59	41.22
Alkali	0.52	30.11	0.99	1.84	0.41	10.51	37.98

## References

[1] Bajpai D, Tyagi VK (2009). Biodiesel: source, production, composition, properties and its benefits. *Journal of Oleo Science*.

[2] Meng X, Yang J, Xu X, Zhang L, Nie Q, Xian M (2009). Biodiesel production from oleaginous microorganisms. *Renewable Energy*.

[3] Chisti Y (2007). Biodiesel from microalgae. *Biotechnology Advances*.

[4] Gouveia L, Oliveira AC (2009). Microalgae as a raw material for biofuels production. *Journal of Industrial Microbiology and Biotechnology*.

[5] Miao X, Li P, Li R, Zhong J (2011). *In situ* biodiesel production from fast-growing and high oil content chlorella pyrenoidosa in rice straw hydrolysate. *Journal of Biomedicine and Biotechnology*.

[6] Makkar HPS, Becker K (2009). Jatropha curcas, a promising crop for the generation of biodiesel and value-added coproducts. *European Journal of Lipid Science and Technology*.

[7] Richmond A (1986). Microalgae of economic potential. *Handbook of Microalgal Mass Culture*.

[8] Haag AL (2007). Algae bloom again. *Nature*.

[9] Huntley ME, Redalje DG (2007). CO_2_ mitigation and renewable oil from photosynthetic microbes: a new appraisal. *Mitigation and Adaptation Strategies for Global Change*.

[10] Pulz O, Gross W (2004). Valuable products from biotechnology of microalgae. *Applied Microbiology and Biotechnology*.

[11] Raja R, Hemaiswarya S, Kumar NA, Sridhar S, Rengasamy R (2008). A perspective on the biotechnological potential of microalgae. *Critical Reviews in Microbiology*.

[12] Park JI, Woo HC, Lee JW (2008). Production of bio-energy from microalgae: status and perspectives. *Korean Journal of Chemical Engineering*.

[13] Matsunaga T, Matsumoto M, Maeda Y, Sugiyama H, Sato R, Tanaka T (2009). Characterization of marine microalga, *Scenedesmus* sp. strain JPCC GA0024 toward biofuel production. *Biotechnology Letters*.

[14] Kim NY, Oh SH, Lee HY, Lee SY (2010). Extraction, purification and property of the lipid from *Scenedesmus* sp.. *Korean Society for Biotechnology and Bioengineering Journal*.

[15] Kim NY, Oh SH, Lee HY, Lee SY (2010). Optimization of lipid extraction from *Scenedesmus* sp. using taguchi approach. *Korean Society For Biotechnology and Bioengineering Journal*.

[16] Ramadhas AS, Jayaraj S, Muraleedharan C (2005). Biodiesel production from high FFA rubber seed oil. *Fuel*.

[17] Georgogianni KG, Kontominas MG, Pomonis PJ, Avlonitis D, Gergis V (2008). Conventional and *in situ* transesterification of sunflower seed oil for the production of biodiesel. *Fuel Processing Technology*.

[18] Carrapiso AI, Timón LM, Petrón JM, Tejeda JF, García C (2000). *In situ* transesterification of fatty acids from Iberian pig subcutaneous adipose tissue. *Meat Science*.

[19] Sahena F, Zaidul ISM, Jinap S (2009). Application of supercritical CO_2_ in lipid extraction—a review. *Journal of Food Engineering*.

[20] Qian J, Wang F, Liu S, Yun Z (2008). *In situ* alkaline transesterification of cottonseed oil for production of biodiesel and nontoxic cottonseed meal. *Bioresource Technology*.

[21] Johnson MB, Wen Z (2009). Production of biodiesel fuel from the microalga schizochytrium limacinum by direct transesterification of algal biomass. *Energy and Fuels*.

[22] Chang M-Y, Tsai G-J, Houng J-Y (2006). Optimization of the medium composition for the submerged culture of Ganoderma lucidum by Taguchi array design and steepest ascent method. *Enzyme and Microbial Technology*.

[23] Mahapatra SS, Patnaik A (2007). Optimization of wire electrical discharge machining (WEDM) process parameters using Taguchi method. *International Journal of Advanced Manufacturing Technology*.

[24] Mohan SV, Rao NC, Prasad KK, Krishna PM, Rao RS, Sarma PN (2005). Anaerobic treatment of complex chemical wastewater in a sequencing batch biofilm reactor: process optimization and evaluation of factor interactions using the taguchi dynamic DOE methodology. *Biotechnology and Bioengineering*.

[25] Ehimen EA, Sun ZF, Carrington CG (2010). Variables affecting the *in situ* transesterification of microalgae lipids. *Fuel*.

[26] Liu B, Zhao Z (2007). Biodiesel production by direct methanolysis of oleaginous microbial biomass. *Journal of Chemical Technology and Biotechnology*.

[27] Quach HT, Steeper RL, Griffin GW (2004). An improved method for the extraction and thin-layer chromatography of chlorophyll a and b from spinach. *Journal of Chemical Education*.

[28] McKittrick BA, Dugar S, ABurnett D Sulfur-substituted azetidinone compounds useful as hypocholesterolemic agents.

[29] Lim YK, Kim DK, Yim ES (2009). Synthesis of biodiesel from vegetable oil and their characteristics in low temperature. *Journal of the Korean Industrial and Engineering Chemistry*.

[30] Müller KD, Husmann H, Nalik HP, Schomburg G (1990). Trans-esterification of fatty acids from microorganisms and human blood serum by trimethylsulfonium hydroxide (TMSH) for GC analysis. *Chromatographia*.

[31] Schuchardt U, Sercheli R, Vargas RM (1998). Transesterification of vegetable oils: a review. *Journal of the Brazilian Chemical Society*.

[32] Encinar JM, González JF, Rodríguez JJ, Tejedor A (2002). Biodiesel fuels from vegetable oils: transesterification of Cynara cardunculus L. Oils with ethanol. *Energy and Fuels*.

[33] Rashid U, Anwar F (2008). Production of biodiesel through optimized alkaline-catalyzed transesterification of rapeseed oil. *Fuel*.

[34] Lam MK, Lee KT, Mohamed AR (2010). Homogeneous, heterogeneous and enzymatic catalysis for transesterification of high free fatty acid oil (waste cooking oil) to biodiesel: a review. *Biotechnology Advances*.

[35] Al-Zuhair S (2007). Production of biodiesel: possibilities and challenges. *Biofuels, Bioproducts and Biorefining*.

[36] Oluwaniyi OO, Ibiyemi SA (2003). Efficacy of catalysts in the batch esterification of the fatty acids of ThevetiaPeruviana seed oil. *Journal of Applied Sciences and Environmental Management*.

[37] Fan X (2008). *Optimization of Biodiesel Production from Crude Cottonseed Oil and Waste Vegetable Oil: Conventional and Ultrasonic Irradiation Methods*.

[38] Taguchi G (1986). *Introduction to Quality Engineering: Designing Quality into Products and Processes*.

[39] Meher LC, Vidya Sagar D, Naik SN (2006). Technical aspects of biodiesel production by transesterification—a review. *Renewable and Sustainable Energy Reviews*.

[40] Dizge N, Aydiner C, Imer DY, Bayramoglu M, Tanriseven A, Keskinler B (2009). Biodiesel production from sunflower, soybean, and waste cooking oils by transesterification using lipase immobilized onto a novel microporous polymer. *Bioresource Technology*.

[41] Xu R, Mi Y (2011). Simplifying the process of microalgal biodiesel production through *in situ* transesterification technology. *Journal of the American Oil Chemists’ Society*.

[42] Wahlen BD, Willis RM, Seefeldt LC (2011). Biodiesel production by simultaneous extraction and conversion of total lipids from microalgae, cyanobacteria, and wild mixed-cultures. *Bioresource Technology*.

[43] Vyas AP, Verma JL, Subrahmanyam N (2010). A review on FAME production processes. *Fuel*.

